# Subcritical Water Extraction of* Chlorella pyrenoidosa*: Optimization through Response Surface Methodology

**DOI:** 10.1155/2018/1931634

**Published:** 2018-11-07

**Authors:** Selvakumar Thiruvenkadam, Shamsul Izhar, Yoshida Hiroyuki, Razif Harun

**Affiliations:** Department of Chemical and Environmental Engineering, Faculty of Engineering, Universiti Putra Malaysia, 43400 Serdang, Malaysia

## Abstract

Subcritical water extraction (SCW) was used to extract oil from* Chlorella pyrenoidosa*. The operational factors such as reaction temperature, reaction time, and biomass loading influence the oil yield during the extraction process. In this study, response surface methodology was employed to identify the desired extraction conditions for maximum oil yield. Experiments were carried out in batch reactors as per central composite design with three independent factors including reaction temperature (170, 220, 270, 320, and 370°C), reaction time (1, 5, 10, 15, and 20 min), and biomass loading (1, 3, 5, 10, and 15%). A maximum oil yield of 12.89 wt.% was obtained at 320°C and 15 min, with 3% biomass loading. Sequential model tests showed the good fit of experimental data to the second-order quadratic model. This study opens the great potential of SCW to extract algal oil for use in algal biofuel production.

## 1. Introduction

The rapid depletion of fossil fuels, together with the uncertain global climate in the past decade, has inevitably led to an increased commercial interest in renewable fuels. Biodiesel is viewed as an attractive potential solution to alleviate the existing dependency on petroleum-based fuels [1]. Current production of biodiesel involves methanolic transesterification of extracted plant lipids, while bioethanol is presently synthesized via anaerobic yeast fermentation of sugar molecules found in the biomass of different food crops [2].

Algae are identified as a promising alternative feedstock for both biofuels due to its high biomass productivity, perceived rapid lipid accumulation, and the suitability of its carbohydrate biochemistry for fermentation process [3]. Additionally, unlike other fuel-producing crops, algae can be grown with saline water in nonagricultural lands, thereby exempting their large-scale cultivation from placing additional demands on precious freshwater and arable lands required for food production [4]. Although algal-based biofuels generate approximately 13% CO_2_ lower emissions from combustion relative to CO_2_ emissions from petroleum diesel [5], in terms of absolute emission levels, algal biofuels can be significantly high for full-scale applications. The development of biofuels from algal biomass has been significantly successful under lab-scale conditions. However, opportunities for commercial-scale applications should focus on addressing related environmental, technological, and economic drawbacks.

The conventional biodiesel production from microalgae has downstream demands such as the moisture content which should not be more than 10%. Since the biochemical products used in the synthesis of biofuel (neutral lipids for biodiesel and simple sugars for bioethanol) are encapsulated within the algal cellular structures, disintegrating the cells to liberate these intracellular products will render them more readily accessible and subsequently enhance production yield. The oil from algae is usually extracted with organic solvent and then converted into biodiesel using a catalyst. The energy intake during drying and solvent extraction processes contributes to two-thirds of the total energy consumption of the entire process [6]. Numerous studies have been reported on different methods available for algal oil extraction [7, 8]. Though these methods were found to be effective in the extraction process, the use of toxic organic solvents, expensive enzymes, and treatment conditions make the process noncommercially feasible. One of the techniques to overcome the existing problem is via Subcritical water extraction (SCW) technology.

SCW has been utilized for solid waste resource recovery and is gaining interest to use in organic reactions due to the fact that water can act as a potential solvent and catalyst. Among the different media used for the reaction, water is attractive because of its safety and low cost. Subcritical states of water are described at a temperature between its boiling point (100°C) and its critical point (374°C) and at pressures high enough to maintain the liquid state. At such conditions, the dielectric constant of water decreases, thereby lowering its polarity. Secondly, the magnitude of ionic product of water increases three orders higher around 250°C compared to at room temperature. These properties are advantageous for the hydrolysis and decomposition of organic compounds including polymeric materials [9]. Extensive studies led by Yoshida and coworkers have concluded that valuable and useful substances, such as organic acids, amino acids, proteins, fatty acids, oils, and nutrition, were made recoverable by utilizing SCW technique for waste treatment. For instance, fish waste was easily liquefied by hydrolysis with SCW, which enabled the recovery of organic acids, amino acids, and the extraction of fatty acids [10]. Similar results were also obtained with squid waste where free fatty acids (FFAs) containing eicosapentaenoic acid (EPA) and docosahexaenoic acid (DHA) were produced during hydrolysis with SCW [11]. Previous studies had shown the characterization of extraction yields from SCW of* Chlorella vulgaris* [12],* Laminaria saccharina* [13], and* Haematococcus pluvialis* [14].

Moreover, SCW provides various advantages over other extraction techniques. The development of an efficient SCW technique for extraction of algal oil is an emerging interest in the biofuel field and creates a novel opportunity to exploit the various valuable properties of extracted oil components. The microalgae strain selected for this study is* Chlorella pyrenoidosa.* This alga was selected to conduct the feasibility study of high biomass productivity low-lipid algal strain for maximum oil production. Hence, the main objective of this work is to study and optimize the effect of process variables such as extraction temperature, extraction time, and biomass loading on oil yield from* C. pyrenoidosa*. Design of Experiment technique accomplishes this objective, i.e., response surface methodology (RSM).

## 2. Materials and Methods

### 2.1. Materials

The microalgae,* Chlorella pyrenoidosa*, was obtained from Sunrise Nutrachem Group Co., Ltd. (Qingdao, China). The powdered microalgal cells were stored inside a desiccator until further used. All solvents were of analytical grade quality and purchased from Sigma Aldrich, Malaysia.

### 2.2. Characterization of Biomass

The moisture content was determined by drying the samples at 105°C and the ash content was determined by incinerating the samples at 550°C. The volatile matter was determined by Thermo-Gravimetric Analyzer (TGA) method. The crude protein and crude fat were estimated using the Kjeldahl method and Soxhlet extraction, respectively. The carbohydrate content was calculated by difference from the total mass of moisture, ash, protein, and fat. The carbon, hydrogen, nitrogen, and sulfur contents of* C. pyrenoidosa* biomass were determined using a CHNS analyzer (model LECO True Spec CHNS628, USA). The oxygen content was calculated by difference from the total mass of carbon, hydrogen, and nitrogen. The higher heating value (HHV) of the alga was calculated using (1) used by Channiwala and Parikh [16]:

(1) HHVMJ/kg=0.3491C+1.1783H+0.1005S−0.1034O−0.0151N−0.0211A

where C, H, N, S, O, and A denotes the mass of carbon, hydrogen, nitrogen, sulfur, oxygen, and ash, on a dry weight basis.

### 2.3. SCW Apparatus and Procedure

**Figure 1:**
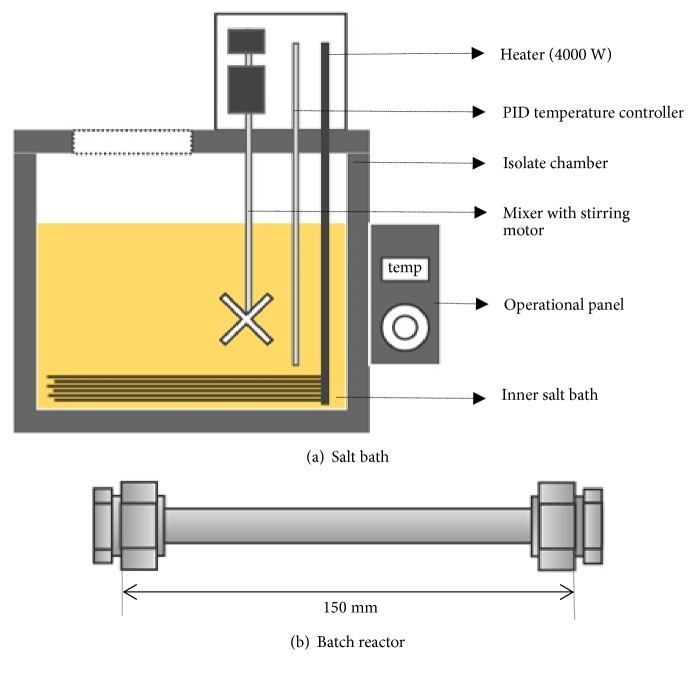
Schematic diagram of the experimental apparatus: (a) salt bath and (b) batch reactor.

The SCW experiments were performed in custom-built stainless-steel reactors of 35 ml capacity, for a total of 20 runs. These batch reactors were assembled from commercially available components (Swagelok Company, Japan) and a schematic view of the reactor setup is illustrated in Figure 1. Milli-Q water was added to the powdered* C. pyrenoidosa* cells to produce wet algae slurry of designated biomass loading according to the experimental runs. In a typical run, about 70% of the algae slurry was loaded into the reactor. The headspace of the reactor was then purged with argon gas to eliminate residual air and thereby, preventing oxidation reactions during the experiments. The reactor was subsequently sealed and immersed in a salt bath (Figure 1) preheated up to the designated reaction temperature. The temperature in the salt bath (Thomas Kogaku Co. Ltd.) was set between 180 and 350°C and for temperature below 180°C, an oil bath (Thomas Kogaku Co. Ltd.) was used. After designated reaction time, the reactors were rapidly cooled to room temperature by quenching them in a water bath.

### 2.4. Product Separation and Recovery

Following the reaction quench, the reactors were carefully disassembled and opened inside a fume hood to vent the gas phase. The gas phase is not examined in this present study due to the use of the batch reactors. The reaction products from the reactor were then transferred to a centrifuge tube. The residual products on the reactor inner wall were recovered on rinsing with distilled water and the recovered water portions were combined to the contents in an existing centrifuge tube. The tubes were then centrifuged at 4000 RPM for 10 min (KUBOTA 2420, Tokyo, Japan). The supernatant and the solid residue were separated after centrifugation. About 1.5 ml of hexane was added to the supernatant containing oil phase and was left for 10 min to facilitate oil-water phase separation. The hexane soluble portion, containing hexane and oil phase, was extracted from the hexane insoluble portion and transferred to the preweighed glass bottle. The hexane washes were repeated until the oil phase was completely recovered. The hexane was removed from the oil phase by evaporation. After hexane evaporation, the glass bottle is weighed, and oil yield is reported.

### 2.5. Characterization of Oil

The free fatty acids (FFAs) in the oil samples were converted to fatty acid methyl esters (FAMEs) by acid transesterification. A fresh solution of methanolic HCl (methanol: concentrated HCl: chloroform, 10:1:1 v/v/v, 3 ml) was added to oil sample for transesterification reaction at 90°C for 60 min. The FAMEs were then extracted and prepared for gas chromatography (GC) analysis following the methods by Lewis* et al.* [17]. The fatty acid compositions in the oil were analyzed using a high-resolution Agilent 6890 Series GC system (Agilent Technologies, USA) equipped with a Zebron capillary column (ZB-WAX, 30 m length, 0.25 mm inner diameter, 0.25 *μ*m film thickness). The oven temperature was first programmed at 100°C hold for 1 min. Then, the temperature was ramped to 230°C at the rate of 5°C/min and maintained for 20 min. The 2 *μ*l sample was injected into the column in a splitless mode. The injector and detector temperatures were 250 and 260°C, respectively. Hydrogen was used as the carrier gas at a flow rate of 3 ml/min with column head pressure at 18 psi.

### 2.6. Statistical Analysis

RSM is a statistical tool, involving different statistically designed combinations, to generate a mathematical model for optimizing the process. The RSM approach using the 3-factor 5-level face-centered central composite design (FCCCD) with 20 experiments was applied to obtain the highest microalgal oil yield from optimizing three most important variables including reaction temperature, reaction time, and biomass loading. The 5 levels of three independent variables used in RSM are given in Table 1. The independent variables are designated as A, B, and C and the dependent variable (response) is designated as Y. The extraction conditions using a combination of independent variables and the response were correlated using a second-order polynomial equation (2):

(2) Y=β0+β1A+β2B+β3C+β12AB+β13AC+β23BC+β11A2+β22B2+β33C2

where *β*_0_ is the model constant coefficient; *β*_1_, *β*_2_, *β*_3_ are linear coefficients; *β*_12_, *β*_13_, *β*_23_ are interaction coefficients; *β*_11_, *β*_22_, *β*_33_ are quadratic coefficients. The analysis of variance (ANOVA) signifies the ratio of a mean square variable due to regression and mean square residual error of each statistically designed combination. All statistical analyses were performed using Design-Expert® 10.0.1 software (Stat-Ease Inc., Minneapolis, USA).

## 3. Results and Discussion

### 3.1. *C. pyrenoidosa* Characterization

The proximate and ultimate characteristics of* C. pyrenoidosa* are shown in Table 2.* C. pyrenoidosa* primarily contained 22.8% carbohydrates, 62.7% proteins, 1.4% lipids, and 5.6% moisture. The biomass was rehydrated to produce wet algae without the addition of any catalysts. It was observed that* C. pyrenoidosa *has high carbon content and higher heating value compared to other freshwater strains reported previously as shown in Table 2.

### 3.2. Response Surface Analysis Based on Central Composite Design

The extraction of oil from algae was optimized through the RSM approach. The combined effect of these variables on the extraction of algal oil was studied with different combinations of the input variables (factors). The experimental design and the results of the oil yield (response) extracted from* C. pyrenoidosa* are listed in Table 3.

The sequential model fitting for the oil extraction was carried out by three different tests: the sequential model sum of squares, lack-of-fit tests, and model summary statistics. Four polynomial models have statistically analyzed the fit summary of the response, namely, linear, interactive (2FI), quadratic, and cubic models, and the results are provided in Table 4. The fit summary output of the response showed that the quadratic model was statistically significant for all the three factors and, therefore, the model has been used for further analysis.

An ANOVA for the response surface quadratic model (Table 5) summarizes the significance of the regression model test, individual model coefficient test, and lack-of-fit test. In the table, “P > F” values less than 0.05 indicate model terms to be significant, while values greater than 0.1000 indicate model terms to be insignificant. In this experiment, as shown in Table 5, the significant model terms are A, C, B^2^, and BC. The model F-value of 15.21 implies the model is significant. There is only a 0.01% chance that a model F-value this large could occur due to noise. The lack-of-fit F-value of 4.43 implies there is a 6.39% chance that a lack-of-fit F-value this large could occur due to noise. The probability P (0.0639) for the lack-of-fit test indicates the adequacy of the model for the observed data at the 95.0% confidence level. The lack-of-fit was observed to be not significant (P > 0.05) and this is suitable to anticipate the response within the limits of factor investigated.

**Figure 2:**
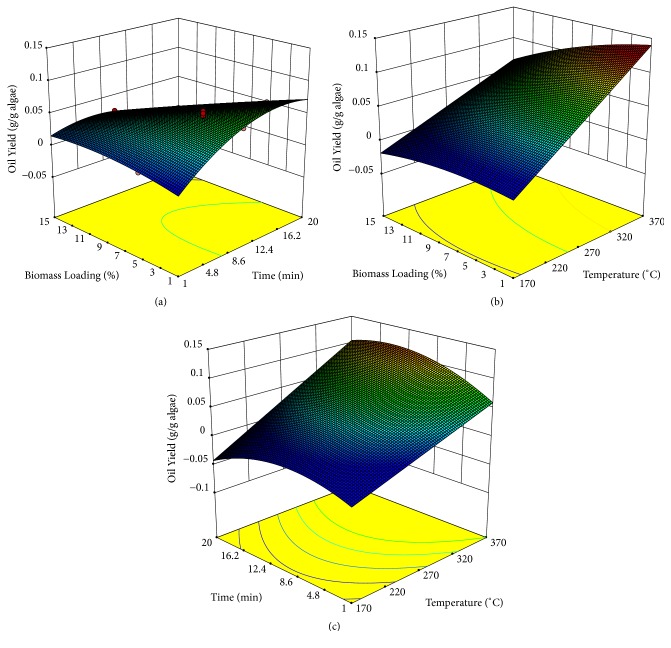
Response surface plots of oil yield (g/g algae) at given (a) reaction temperature (°C), (b) reaction time (min), and (c) biomass loading (%).

The regression coefficients and ANOVA of the predicted quadratic model to the response oil yield are given in Table 5. The positive and negative value of regression coefficients indicate the synergistic and antagonistic effects, respectively. The degree of correlation between the observed and predicted values is indicated by the R^2^ value of the model. In this case, the R^2^ value of the quadratic model for oil yield was 0.9319, which explains that 0.0681% of the total variations in the oil yield were not explained by the model and thereby, the fitted quadratic model is suitable. The oil yield was influenced by reaction temperature and biomass loading, both the linear terms, whereas reaction time was affected only through its quadratic terms. The ANOVA table suggested that the linear terms as most significant terms (81%) were based on the sum of squares. Quadratic terms (12.5%) and interaction terms (6.4%) exhibited the lowest level of significance. The three-dimensional surface plots (Figure 2) display the interactions in response surface plots between the response and two factors when the third variable being constant. The empirical relationship between three independent factors and the response was expressed by the following second-order polynomial model equation (3):

(3) Y=0.054+0.058A−0.021C+0.015AB−0.016AC−0.029BC−0.029B2

where Y is the oil yield; A is the reaction temperature; B is the reaction time; and C is the biomass loading.

The maximum oil yield of 12.68 wt.% was extracted at the following SCW conditions: 320°C reaction temperature, 15 min reaction time, and 3% biomass loading. The chromatography analysis of this oil sample, extracted at 320°C, was highlighted on eight FAMEs as presented in Table 6. Our observations regarding the fatty profiling showed that monounsaturated fatty acids (MUFAs) were more in abundant. The most common FAMEs in biodiesel are methyl palmitate (C16:0), methyl stearate (C18:0), methyl oleate (C18:1), methyl linoleate (C18:2), and methyl linolenate (C18:3) [18]. In our study, methyl palmitate (C16:0) and methyl palmitoleate (C16:1) were abundant in the oil extracted at 270°C and 320°C, respectively. Higher contents of unsaturated FAMEs in oil extracted at temperatures above 320°C is an indicator of the production of high-quality biodiesel.

### 3.3. Effect of Process Variables on Oil Yield

#### 3.3.1. Effect of Temperature

**Figure 3:**
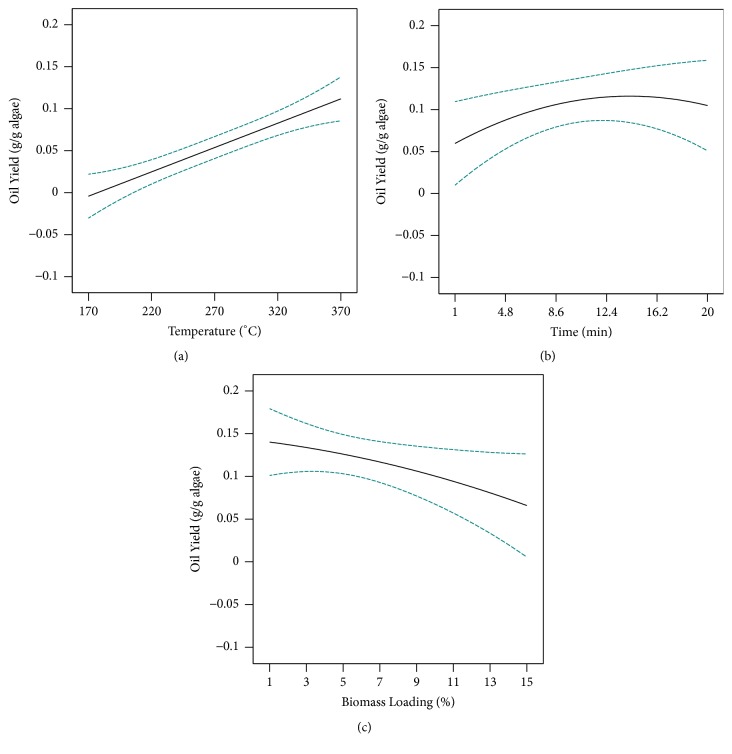
The effect of (a) reaction temperature, (b) reaction time, and (c) biomass loading on the oil yield.

The main factor affecting oil yield was the reaction temperature. In the present study, the effect of temperature on oil extraction was investigated for five different temperatures: 170°C, 220°C, 270°C, 320°C, and 370°C. A significant difference (P < 0.05) was observed between the tested temperatures in terms of oil yield from the statistical analysis. The oil yield increased with the increase of temperature from 170°C to 320°C, resulting in the maximum response for oil yield occurring at 320°C. With further increase in temperature, the oil yield was considerably decreased. Based on the results shown in Table 5, it can be concluded that the extraction temperature exhibits a positive linear and positive quadratic effect on oil yield.  Figure 3(a) shows the effect of temperature on the oil yield and it could be observed that the oil yield is directly proportional to the reaction temperature. Increasing the reaction temperature resulted in increased oil yield, primarily at lower biomass loading (Figure 2(b)). Reaction temperatures up to 300°C, which enhance the thermal degradation of organic components into the oil phase, result in increased oil yields. However, a further increase in temperature induces the formation of char from the polymerization of thermo-sensitive oil intermediates and decreases the oil yield [19]. According to the experimental results of the hydrothermal liquefaction of* C. pyrenoidosa* obtained by Gai* et al.*, it was observed that an increase in temperature from 260 to 280°C led to an increased oil yield. However, it was also found that with further increase in temperature from 280 to 320°C decreased oil yield due to the formation of gaseous products. The results are in agreement with Shuping* et al.* [20], Chen* et al.* [21], Gai* et al.* [22], and Anastasakis and Ross [14].

#### 3.3.2. Effect of Reaction Time

The effect of reaction time on the oil yield was studied by carrying out SCW reactions from 1 to 20 min. The oil yield increased with the increase in reaction time where the increased being reached at lower biomass loading. This trend is clearly observed in the curves shown in Figures 2(a) and 2(c); furthermore, this explains the positive linear and negative quadratic effects of reaction time.  Figure 3(b) shows the effect of the reaction time on the oil yield. The oil yield increases when the reaction time is increased from 1 to 15 min; subsequently, the oil yield gradually decreased between 15 and 20 min of reaction time. During coliquefaction of microalgae* C. pyrenoidosa* and rice husk, Gai* et al.* [19] discovered a consistent increase in oil yield up to 60 min reaction time and with increasing reaction time, oil yield decreased due to further recondensation or repolymerization of oil products. In a study to investigate the effect of residence time on SCW of* L. saccharina*, 15 min was found to be the optimum holding time at 350°C and further increase in residence time decreased biocrude yield due to subsequent condensation and/or polymerization of biocrude intermediates to form new high molecular weight products [14]. This was supported by Xu* et al.* [23], Jin* et al.* [24], and Shuping* et al.* [20], who observed decreasing in biocrude yield beyond the threshold points 30 min, 40 min, and 50 min, respectively. Also, a short residence time during SCW increases biocrude yield due to the rapid release of the intracellular components of the biomass [25]. A short residence time is beneficial to reduce the costs for commercial-scale applications involving small reactors.

#### 3.3.3. Effect of Biomass Loading

Figure 3(c) shows the effect of biomass loading on the oil yield. The oil yield is inversely proportional to biomass loading in this study. Lower biomass loading contributes to higher oil yield, at higher temperatures and longer reaction times. The surface plots in Figures 2(b) and 2(c) also confirmed the observed effect of biomass loading on the oil yield. However, coliquefaction of microalgae* C. pyrenoidosa* and rice husk showed that, with the increased solid concentration from 10 to 30 wt.%, the yields of biocrude oil increased slightly then decreased, and the highest yield was achieved at 20 wt.% [19]. In the hydrothermal environment, water acts both as a medium of heat-transfer and as a reactant of a hydrogen donor. According to Akhtar and Amin [26], the increased solid concentration may inhibit the interactions between molecules of biomass and water, suppressing the dissolution of the biomass components. Thus, the yield of biocrude oil decreased at higher solid concentration.

### 3.4. Process Optimization

For optimization, we set the target for each factor and the response. Based on the economic perspective of the production process, the reaction temperature was set to minimize while the other factors were set within the studied range and goals are targeted to achieve maximum possible yield by the software. The optimum conditions, namely, reaction temperature of 277°C, the reaction time of 16 min, and biomass loading of 1% corresponding to a maximum oil yield of 8.29 wt.%, were predicted through RSM. The suitability of the model equation for predicting the maximum response value was tested experimentally using the recommended optimum conditions. The experimental value (7.96 wt.%) obtained was in good agreement with the predicted results of the software.

## 4. Conclusion

This study demonstrated the extraction of oil from low-lipid algae species via subcritical water extraction. The oil yield was optimized using a central composite design based on response surface methodology. The three independent variables, namely, reaction temperature, reaction time, and biomass loading, significantly influenced the extraction yield of the oil. The maximum oil yield (12.68 wt.%) is obtained with the combination of 320°C reaction temperature, 15 min reaction time, and 3% biomass loading. The mathematical model derived based on multiple response optimization represents the process conditions to obtain an optimum response. Economically viable subcritical water extraction of algae oil could find promising potential in the biofuel sector.

## Figures and Tables

**Table 1:** Levels of independent variables used for optimization.

Independent variables	Levels				
A: Reaction temperature (°C)	170	220	270	320	370
B: Reaction time (min)	1	5	10	15	20
C: Biomass loading (%)	1	3	5	10	15

**Table 2:** Proximate, ultimate, and biochemical analysis of freshwater algae species.

Properties	*Chlorella pyrenoidosa*	*Oedogonium *sp.	*Cladophora vagabunda*
*Proximate (wt.%)*			
Moisture	5.60	6.5	5.7
Ash	7.50	20.6	17.8

*Biochemical (wt.%)*			
Organic content	86.90	72.9	76.5
Carbohydrate	22.80	41.0	44.4
Protein	62.70	22.5	26.8
Lipid	1.40	9.4	5.3

*Ultimate (wt.%)*			
Carbon	44.53	36.6	37.5
Hydrogen	5.71	5.7	5.9
Oxygen	38.87^a^	30.9	32.9
Nitrogen	9.80	4.8	6.5
Sulfur	1.09	0.4	1.8
HHV (MJ/kg)	18.06	15.8	16.4
Reference	This study	[15]	[15]

^a^O (wt.%) = 100 – (C + H + N) (wt.%); HHV: higher heating value.

**Table 3:** Response values of the oil yield for given levels of variables (reaction temperature, reaction time, and biomass loading) in response surface methodology.

Run Number	Type	Independent variables			Dependent variable
		Reaction temperature, A (°C)	Reaction time, B (min)	Biomass loading, C (%)	Oil Yield, Y (g/g algae)
1	Factorial	320	5	10	0.0702
2	Factorial	320	15	3	0.1268
3	Factorial	320	5	3	0.0742
4	Axial	270	10	15	0.0250
5	Center	270	10	5	0.0659
6	Center	270	10	5	0.0497
7	Center	270	10	5	0.0624
8	Axial	170	10	5	0.0101
9	Factorial	220	15	3	0.0173
10	Factorial	220	5	3	0.0053
11	Axial	270	10	1	0.0625
12	Axial	370	10	5	0.1091
13	Factorial	220	5	10	0.0053
14	Factorial	320	15	10	0.0683
15	Axial	270	1	5	0.0118
16	Center	270	10	5	0.0543
17	Factorial	220	15	10	0.0115
18	Center	270	10	5	0.0585
19	Axial	270	20	5	0.0506
20	Center	270	10	5	0.0697

**Table 4:** Selection of a suitable model for the SCW system (fit summary).

Source	Sum of squares	d.f.	Mean square	F	P > F	Remark
**Sequential model sum of squares**						
Mean	0.051	1	0.051			
Linear	0.018	3	6.105E-003	26.62	< 0.0001	Suggested
2FI	7.103E-004	3	2.368E-004	1.04	0.4075	
Quadratic	1.463E-003	3	4.877E-004	3.26	0.0680	Suggested
Cubic	1.221E-003	5	2.443E-004	4.43	0.0639	Aliased
Residual	2.754E-004	5	5.509E-005			
Total	0.073	20	3.642E-003			
**Lack-of-fit tests**						
Linear	3.395E-003	11	3.086E-004	5.60	0.0350	Suggested
2FI	2.684E-003	8	3.356E-004	6.09	0.0311	
Quadratic	1.221E-003	5	2.443E-004	4.43	0.0639	Suggested
Cubic	0.000	0				Aliased
Pure Error	2.754E-004	5	5.509E-005			
**Model Summary statistics**						
*Source*	*S.D.*	*R* ^*2*^	*Adj. R* ^*2*^	*Pre. R* ^*2*^	*PRESS*	*Remark*
Linear	0.015	0.8331	0.8018	0.7157	6.251E-003	Suggested
2FI	0.015	0.8654	0.8032	0.5914	8.984E-003	
Quadratic	0.012	0.9319	0.8707	0.5038	0.011	Suggested
Cubic	7.422E-003	0.9875	0.9524			Aliased

**Table 5:** ANOVA for the regression model for the prediction of oil yield.

Source	Coefficient estimate	d.f.	Standard error	Sum of squares	Mean square	F-value	P-value	Remark
Model		9		0.020	2.277E-003	15.21	0.0001	Significant
A	0.058	1	8.154E-003	7.563E-003	7.563E-003	50.53	< 0.0001	
B	7.131E-003	1	7.782E-003	1.257E-004	1.257E-004	0.84	0.3811	
C	-0.021	1	5.944E-003	1.912E-003	1.912E-003	12.77	0.0051	
A^2^	1.794E-005	1	9.764E-003	5.055E-010	5.055E-010	3.378E-006	0.9986	
B^2^	-0.029	1	9.665E-003	1.387E-003	1.387E-003	9.27	0.0124	
C^2^	-8.669E-003	1	0.011	1.011E-004	1.011E-004	0.68	0.4304	
AB	0.015	1	0.016	1.322E-004	1.322E-004	0.88	0.3695	
AC	-0.016	1	0.017	1.379E-004	1.379E-004	0.92	0.3597	
BC	-0.029	1	0.016	4.930E-004	4.930E-004	3.29	0.0996	
Residual		10		1.497E-003	1.497E-004			
Lack of fit		5		1.221E-003	2.443E-004	4.43	0.0639	Not significant
Pure error		5		2.754E-004	5.509E-005			
Cor total		19		0.022				
Adeq. Prec.	14.788							

**Table 6:** Major compounds in biocrude oil from* C. pyrenoidosa *at different reaction temperatures.

Order	Retention time (min)	Fatty acid methyl esters (FAMEs)	FAME content (%)				
			170°C	220°C	270°C	370°C	370°C
1	6.06	Methyl myristoleate (C14:1)			38.41	4.44	1.97
2	8.78	Methyl palmitate (C16:0)			61.59	5.41	
3	9.00	Methyl palmitoleate (C16:1)				69.03	
4	12.13	Methyl oleate (C18:1)					0.90
5	12.21	Methyl vaccenate (C18:1)				2.54	
6	13.68	Methyl linolenate (C18:3)				8.67	
7	15.20	Methyl 11-eicoenoate (C20:1)		9.35			
8	15.78	Methyl 11-14 eicosadienoate (C20:2)					62.71
9	16.42	Methyl arachidonate (C20:4)					2.91
10	16.68	Methyl 11-14-17 eicosapentaenoate (C20:3)					7.14
11	17.42	Methyl eicosapentaenoate (C20:5)				4.11	3.91
12	18.11	Methyl erucate (C22:1)					0.90
13	18.81	Methyl lignocerate (C24:0)	100	90.65			0.76
14	20.66	Methyl docosahexaenoate (C22:6)				1.61	3.75
15	20.93	Methyl nervonate (C24:1)				4.19	15.05

		Saturated fatty acids (%)	100	90.65	61.59	5.41	0.76
		Monounsaturated fatty acids (%)		9.35	38.41	80.20	18.82
		Polyunsaturated fatty acids (%)				14.39	80.42

## Data Availability

The data used to support the findings of this study are available from the corresponding author upon request.
